# 106. High Maternal Tdap Vaccine Uptake During Early Part of Vaccination Window: Implications for Future Maternal Vaccines

**DOI:** 10.1093/ofid/ofac492.184

**Published:** 2022-12-15

**Authors:** Amy W Law, Jennifer Judy, Sarah J Willis, Kimberly M Shea

**Affiliations:** Pfizer, Inc., New York, New York; Pfizer, New York, New York; Pfizer, New York, New York; Pfizer Inc., Newton, Massachusetts

## Abstract

**Background:**

Maternal vaccines to prevent respiratory syncytial virus (RSV) among infants are in development. Uptake of existing maternal vaccines can be used to predict uptake of future maternal RSV vaccines and may be used to inform vaccine policy decisions. Previous reports of maternal vaccination rates do not estimate vaccine uptake by gestational week (wGA) of pregnancy, which is needed for precise estimation of vaccine impact. This study estimated the uptake of maternal Tdap vaccination overall and by wGA in a large electronic health records (EHR) database representing both privately and publicly insured patients over a recent 5-year period.

**Methods:**

We identified pregnant women aged 15 – 44 years who had a live birth delivery between 01/01/2017 – 9/29/2021 in the Optum EHR database. Continuous activity for 6 months pre-conception through 1 day after delivery were required. Patients with >1 type of pregnancy outcome within 7 days and/or unidentifiable wGA were excluded. We utilized recently published gestational age algorithms to estimate the uptake of maternal Tdap vaccination overall and by wGA of pregnancy. Results were reported by year.

**Results:**

The population included 1,056,488 live births among 919,510 pregnant women during the study period. The average age at delivery was 29.7 years (SD: 5.6), 72% were white, 82% were non-Hispanic; 58% had private insurance, and 38% had Medicaid. Overall, 56% of the pregnancies included a Tdap vaccine during their pregnancy. Among vaccinated pregnancies, the majority (68%) of Tdap vaccines were administered during the first 6 weeks of the recommended 10-week vaccination window (CDC recommends Tdap vaccination from 27-36 wGA) (**Table)**.

**Table.**
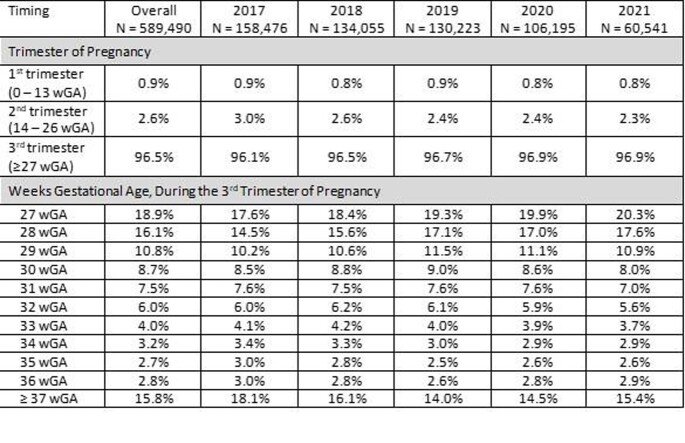
Timing of Maternal Tdap Vaccination among Pregnant Women, by Year

**Conclusion:**

In this analysis using a large EHR database, the overall uptake of maternal Tdap vaccination was consistent with previously published estimates. Notably, the majority of Tdap vaccination occurred during the earliest weeks of the recommended vaccination period. These results may have important implications for estimating potential impact of future maternal vaccines.

**Disclosures:**

**Amy W. Law, PharmD**, Pfizer: Employment|Pfizer: Stocks/Bonds **Jennifer Judy, MS, PhD**, Pfizer Inc: Employee|Pfizer Inc: Stocks/Bonds **Sarah J. Willis, PhD, MPH**, Pfizer: Pfizer supported research at Harvard Pilgrim Health Care Institute (paid to Institute)|Pfizer: Employment **Kimberly M. Shea, Ph.D., M.P.H.**, Pfizer: Employee|Pfizer: Stocks/Bonds.

